# Inhibiting DHN- and DOPA-melanin biosynthesis pathway increased the therapeutic value of itraconazole in *Madurella mycetomatis* infected *Galleria mellonella*

**DOI:** 10.1093/mmy/myac003

**Published:** 2022-01-22

**Authors:** Wilson Lim, Mickey Konings, Florianne Parel, Kimberly Eadie, Nikolaos Strepis, Ahmed Fahal, Annelies Verbon, Wendy W J van de Sande

**Affiliations:** Erasmus MC, University Medical Center Rotterdam, Department of Medical Microbiology and Infectious Diseases, Rotterdam, the Netherlands; Erasmus MC, University Medical Center Rotterdam, Department of Medical Microbiology and Infectious Diseases, Rotterdam, the Netherlands; Erasmus MC, University Medical Center Rotterdam, Department of Medical Microbiology and Infectious Diseases, Rotterdam, the Netherlands; Erasmus MC, University Medical Center Rotterdam, Department of Medical Microbiology and Infectious Diseases, Rotterdam, the Netherlands; Erasmus MC, University Medical Center Rotterdam, Department of Medical Microbiology and Infectious Diseases, Rotterdam, the Netherlands; Mycetoma Research Centre, University of Khartoum, Khartoum, Sudan; Erasmus MC, University Medical Center Rotterdam, Department of Medical Microbiology and Infectious Diseases, Rotterdam, the Netherlands; Erasmus MC, University Medical Center Rotterdam, Department of Medical Microbiology and Infectious Diseases, Rotterdam, the Netherlands

**Keywords:** mycetoma, eumycetoma, melanin, neglected tropical disease, Madurella mycetomatis

## Abstract

Eumycetoma is a neglected tropical disease, and *Madurella mycetomatis*, the most common causative agent of this disease forms black grains in hosts. Melanin was discovered to be one of the constituents in grains. Melanins are hydrophobic, macromolecular pigments formed by oxidative polymerisation of phenolic or indolic compounds. *M. mycetomatis* was previously known to produce DHN-melanin and pyomelanin *in vitro.* These melanin was also discovered to decrease *M. mycetomatis*’s susceptibility to antifungals itraconazole and ketoconazole *in vitro*. These findings, however, have not been confirmed *in vivo.* To discover the melanin biosynthesis pathways used by *M. mycetomatis in vivo* and to determine if inhibiting melanin production would increase *M. mycetomatis's* susceptibility to itraconazole, inhibitors targeting DHN-, DOPA- and pyomelanin were used. Treatment with DHN-melanin inhibitors tricyclazole, carpropamid, fenoxanil and DOPA-melanin inhibitor glyphosate in *M. mycetomatis* infected *Galleria mellonella* larvae resulted in presence of non-melanized grains. Our finding suggested that *M. mycetomatis* is able to produce DOPA-melanin *in vivo*. Inhibiting DHN-melanin with carpropamid in combination with the antifungal itraconazole also significantly increased larvae survival. Our results suggested that combination treatment of antifungals and melanin inhibitors can be an alternative treatment strategy that can be further explored. Since the common black-grain eumycetoma causing agents uses similar melanin biosynthesis pathways, this strategy may be applied to them and other eumycetoma causative agents.

**Lay Summary:**

Melanin protects fungi from environmental stress and antifungals. We have discovered that *Madurella mycetomatis* produces DHN-, pyomelanin and DOPA-melanin *in vivo*. Inhibiting *M. mycetomatis* DHN-melanin biosynthesis increases therapeutic value of the antifungal itraconazole *in vivo*.

## Introduction

Mycetoma is a subcutaneous implantation infection recognized in 2016 as a neglected tropical disease by the World Health Organisation.^1–3^ Eumycetoma is the most common type of mycetoma and is characterized by large tumor-like swellings, sinus formation discharging grains and is frequently located in the extremities.^4,5^ This disease can be caused by many different fungal species, with the most common causative agent being the fungus *Madurella mycetomatis.*^2^ Most cases occur in the mycetoma belt in the tropical and the sub-tropical regions in the world.^6^

Eumycetoma is most often treated with the regimen used by the Mycetoma Research Centre in Sudan where it consists of 400 mg/day itraconazole for the first 6 months, surgical removal of the lesion, followed by another course of 400 mg/day itraconazole for at least 6 more months.^7^ However, treatment duration is often longer and still, a favorable treatment outcome may not be achieved. Although *M. mycetomatis* is very susceptible towards itraconazole and ketoconazole *in vitro*, the cure rates of these antifungals in mycetoma patients are disappointing.^8,9^ The cure rate of itraconazole is generally low between 8 and 26%, with a postoperative recurrence rate between 25 and 50%, and an amputation rate of 2.8%.^7,10,11^ Treatment with ketoconazole resulted in a cure rate of 56.1% and an amputation rate of 6.1%.^10^ The low *in vivo* efficacy of itraconazole could be the result of the formation of protective structures *in vivo.* In mycetoma, the causative agent forms grains in the infected tissue. These grains come in different textures, colors and consistency depending on the causative agent.^12^ Grains formed by *M. mycetomatis* are black and consist of a densely packed fungal mycelium embedded in a hard, brown matrix composed of an extra-cellular cement material.^12,13^ Although the exact nature of this cement material is currently unknown, melanin was demonstrated to be one of its many constituents.^12,13^

Melanins are hydrophobic, negatively charged, macromolecular pigments formed by oxidative polymerization of phenolic or indolic compounds.^12^ They protect fungi against environmental stress, antifungal agents and host defences.^14–18^ We have previously demonstrated that *M. mycetomatis* melanin offered protection against strong oxidants and the antifungal agents itraconazole (ITZ) and ketoconazole *in vitro.*^12^ This effect was also reported for chromoblastomycosis causative agents *Fonsecaea* spp. It is therefore very likely that the presence of melanin in *M. mycetomatis* grain contributes to its poor response to antifungal agents. In fungi, melanin can be synthesized via various biosynthetic pathways which differ from those in humans.^19–21^*Aspergillus fumigatus, Aspergillus nidulans* and *M. mycetomatis* synthesize melanin from acetyl-coenzyme A via the polyketide pathway to form 1,8-dihydroxynaphthalene (DHN) melanin.^15,22^*Cryptococcus neoformans* produces melanin via oxidation of L-3,4-dihydroxyphenylalanine (L-DOPA) in the presence of laccase.^22,23^ Both DHN- and DOPA-melanin are cell wall bound. A third fungal melanin, pyomelanin, is often found to be excreted and is synthesized via tyrosine degradation pathway through homogentisic acid. Pyomelanin is also produced in *A. fumigatus* and *M. mycetomatis*, indicating that more than one type of melanin can be synthesized in several fungi.^12,14^

**Figure 1.**
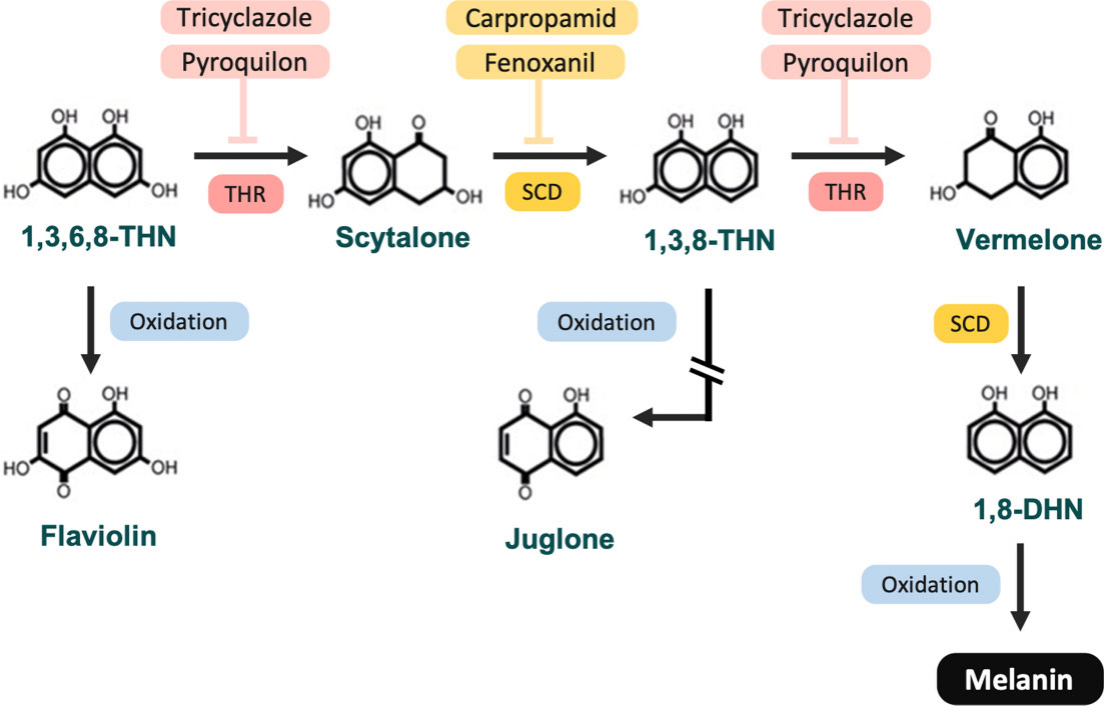
DHN-melanin biosynthesis pathway. Tricyclazole and pyroquilon inhibits tetrahydroxynaphthale reductase (THR) while carpropamid and fenoxanil inhibits scytalone dehydratase (SCD) in the DHN-melanin pathway. Flaviolin and Juglone are by products that are generated when DHN-melanin is inhibited by tricyclazole and pyroquilon.

Since fungal melanin pathways are not present in humans, they are a good target for antifungal drugs. Although not used in human medicine, inhibitors of fungal melanin pathways are used as fungicide in agriculture.^24–29^ Melanin inhibitors Tricyclazole (TCZ) and pyroquilon (PYR) inhibit tetrahydroxynaphthalene reductase (THR) in the DHN-melanin biosynthetic pathway (Fig. 1), thereby interfering with the production of scytalone and vermelone. Carpropamid (CAR) and fenoxanil (FNX) inhibits scytalone dehydratase (SCD), the single dehydratase operating within the DHN melanin biosynthesis pathway by inhibiting the production of 1,3,8–Trihydroxynaphthalene reductase (Fig. 1).^24,30–33^ Sulcotrione (SCT) inhibits 4-hydroxyphenylpyruvate dioxygenase in the pyomelanin pathway, while glyphosate (GLY) acts on the shikimate pathway by inhibiting the enzyme 5-enolpyruvoylshikimate 3-phosphatesynthase (EPSPS), inhibiting DOPA-melanin production.^12,34,35^

We have previously demonstrated that TCZ and SCT inhibited DHN- and pyomelanin production in *M. mycetomatis in vitro.*^12^ The same melanin inhibitors were also used to determine melanin biosynthesis pathways in other black grain eumycetoma causative agents.^36^ TCZ, GLY and SCT inhibited DHN-, DOPA- and pyomelanin production in *Medicopsis romeroi* and *Falciformispora senegalensis* while SCT inhibited pyomelanin production in *Trematosphaeria grisea* and *Falciformispora tompkinsii in vitro.*^36^ However, their ability to inhibit melanin biosynthesis in grains has not yet been determined. We have since developed an *in vivo M. mycetomatis* grain model in the *Galleria mellonella* larvae which forms black melanized grains resembling those found in human.^37^ Here, we determine if the melanization of *M. mycetomatis* grains *in vivo* could be inhibited and also determine if non-melanized *M. mycetomatis* grains are more susceptible to itraconazole treatment.

## Methods

### Fungal isolates

The *M. mycetomatis* reference genome strain MM55 was used in all *in vivo G. mellonella* experiments.^38^ MM55 was originally obtained from the Mycetoma Research Center in Sudan and maintained in Erasmus Medical Centre, the Netherlands. Isolates were maintained in the laboratory on Sabouraud Dextrose Agar (Difco laboratories, Becton and Dickinson, Sparks, USA) at 37ºC and identified to the species level on the basis of morphology, polymerase chain reaction (PCR)-based restriction fragment length polymorphisms, and sequencing of the ITS region.^39,40^

### Melanin inhibitors

Melanin inhibitors; Carpropamid (CAR), Fenoxanil (FNX), Glyphosate (GLY), Pyroquilon (PYR), Sulcotrione (SCT) and Tricyclazole (TCZ) were obtained from Sigma-Aldrich (Sigma-Aldrich Chemie NV, Netherlands) and reconstituted in DMSO. Juglone, a DHN-intermediate product, was also obtained from Sigma-Aldrich and reconstituted in DMSO. Melanin inhibitors were diluted in PBS and administered into the larvae at 5 and 50 mg/kg with a final concentration of DMSO at 5%. This DMSO concentration does not result in toxicity in the larvae. TCZ, CAR, SCT and GLY could only be tested at 5 mg/kg and not at higher concentrations due to low solubility at high concentrations.

### Toxicity of melanin inhibitors in *G. mellonella* larvae

To determine the toxicity of the melanin inhibitors and juglone (a DHN-intermediate product in *G. mellonella*), larvae were injected with 5 or 50 mg/kg of melanin inhibitors and 0.4, 4 and 40 μg/larvae of juglone for three consecutive days in different proleg with an 29G U-100 insulin needle (BD Diagnostics, EU). Controls were injected with PBS only. Toxicity studies were performed with groups of 15 larvae and larvae survival was monitored for ten days. A non-significant difference in larvae survival between the treated and the control groups indicated a lack of noticeable toxicity up to the dosage administered.^37^

### Infection of *G. mellonella* larvae with *M. mycetomatis* and melanin inhibitors

Final sixth instar *G. mellonella* larvae were acquired from SA.G.IP (Bagnacavallo, Italy). They were kept at room temperature on wood shavings in the dark and used within 5 days of receipt. Larvae of approximately 300 to 500 mg showing no discoloration were selected for use in experiments. To determine the therapeutic value of these melanin inhibitors, G. *mellonella* larvae were infected with *M*. *mycetomatis* isolate MM55 according to a previously published protocol.^37^ In short, *M*. *mycetomatis* mycelia were cultured in colorless RPMI 1640 medium supplemented with L-glutamine (0.3 g/l), 20 mM morpholinepropanesulfonic acid (MOPS) and chloramphenicol (100 mg/l; Oxoid, Basingstoke, United Kingdom) for 2 weeks at 37°C. The fungal culture was then filtered through a 22 μM filter (Whatman) and then sonicated for 2 min at 10 microns (Soniprep, Beun de Ronde, The Netherlands). The resulting homogenous suspension was then harvested and adjusted to a concentration of 1 g/10 ml. Inoculation was performed by injecting 40 μl of the fungal suspension into the last left pro-leg with a 29G U-100 insulin needle (BD diagnostics, Sparsk, USA) resulting in an inoculum size of 4 mg wet weight per larva. Infected larvae were administered with DHN inhibitors TCZ (5 mg/kg), PYR (50 and 5 mg/kg), CAR (5 mg/kg) or FNX (5 mg/kg), pyomelanin inhibitor SCT (5 mg/kg) or DOPA melanin inhibitor GLY (5 mg/kg) 2 h prior to infection, 22 h after infection and 46 h after infection the on different larvae prolegs with insulin needles. Itraconazole was administered at 5.7 mg/kg larvae as previously done.^37^ Controls were injected with PBS. Infection studies were performed with groups of 15 larvae and larvae survival was monitored for 10 days. Pupa that are formed during these 10 days were left out of the equation while blackened and deflated larvae unresponsive to external physical stimuli is considered dead. Investigation was performed in triplicates.

### Hemolymph melanization

Melanization is a part of the larvae's immune reaction in response to invading pathogens. To determine the melanization of *G. mellonella* after infection with *M. mycetomatis* and treatment with melanin inhibitors, larvae hemolymph was harvested at 24 h post-infection and measured at 405 nm as previously described.^37^ To harvest hemolymph, a small incision was first made below the last proleg of the larvae with a scalpel. Hemolymph were then squeezed out into sterile 1.5 ml tubes and diluted 1:10 with IPS buffer (Insect Physiological Saline: 150 mM sodium chloride, 5 mM potassium chloride, 10 mM Tris-HCl pH 6.9, 10 mM EDTA and 30 mM sodium citrate). Each hemolymph sample was measured in triplicates.

### Histology

To visually observe the difference in grains formed by *M. mycetomatis* in *G. mellonella* in the presence of melanin inhibitors, four to five larvae from each group were sacrificed on the third day post infection, fixed in 10% buffered formalin, dissected longitudinally into two halves with a scalpel and processed for histology. Sections were stained with hematoxylin and eosin (HE) and Grocott methanamine silver. Grains were magnified 40x and visualized on the computer screen using the supplied EOS Utilitysoftware (Canon Inc). Grains were categorized into large, medium or small sizes using the enlargement display frame present in the Live View Shooting mode and manually counted under a light microscope mounted with a Canon EOS70D camera (Canon Inc.) by two independent scientists as previously described.^41^ The sum of all large, medium and small grains present in larvae was used to represent the total number of grains in the larvae. To estimate the total size of grain present in the larvae, the sum of all grains in a larva was multiplied by the minimum size of their respective category (large: 0.02 mm^2^, medium: 0.01 mm^2^ and small: 0.005 mm^2^).

### Statistical analysis

To compare survival curves, the Log-rank test was performed with GraphPad Prism 7 (GraphPad Inc.). To determine the statistical difference in the total number and sizes of grains between the treated and non-treated groups, a Mann-Whitney test was performed with GraphPad Prism 7. A *P*-value smaller than .05 was deemed significant.

## Results

### Melanin inhibitors are able to inhibit melanization of *M. mycetomatis* grains *in vivo*

**Figure 2.**
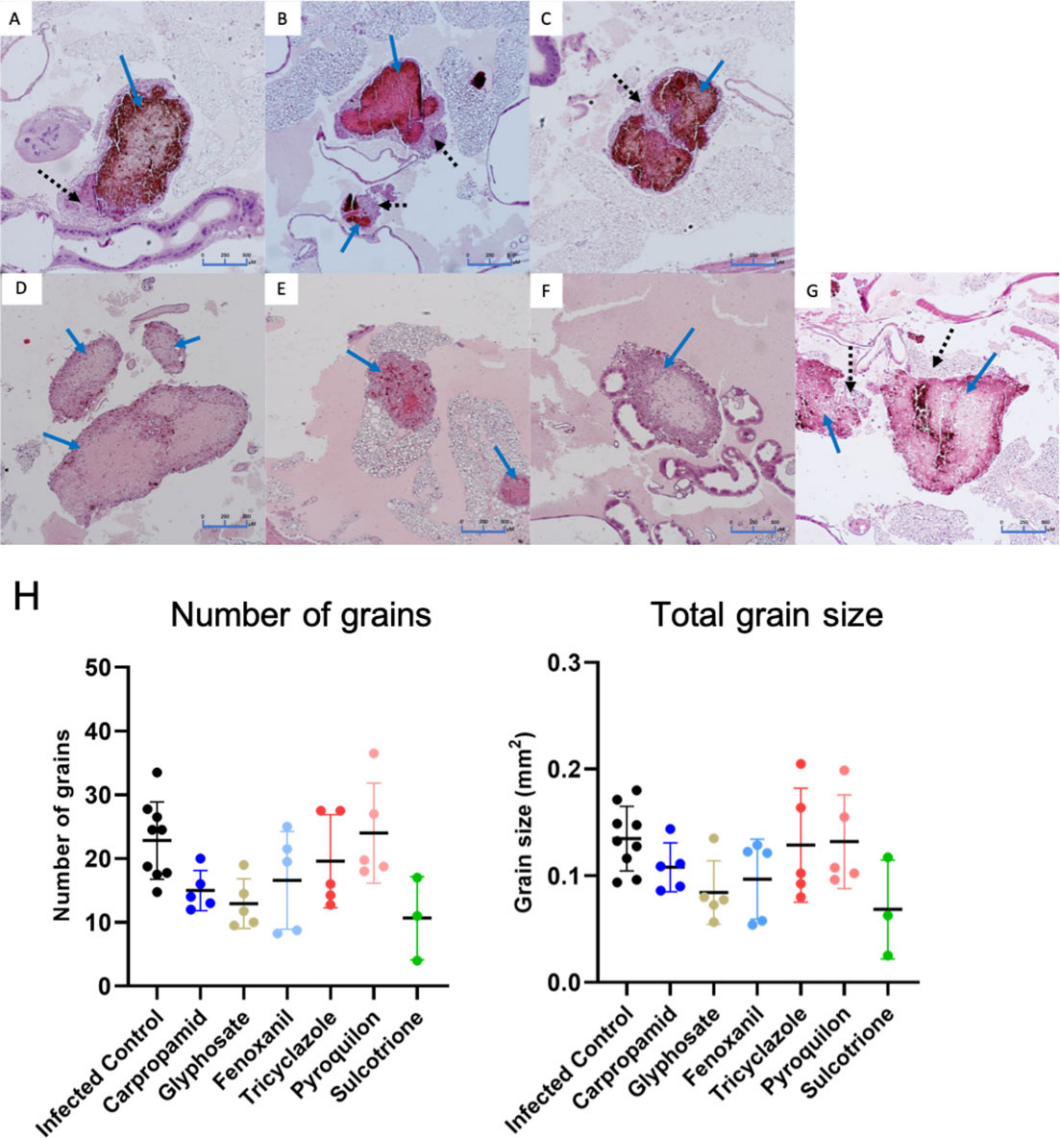
Fungal burden in *G*. *mellonella* larvae infected with *M*. *mycetomatis* and treated with melanin inhibitors. Histopathological sections of larvae treated with different melanin inhibitors and sacrificed 72 h after inoculation stained with H&E to demonstrate the presence of fungal grains and encapsulation. Panel A, Infected control group treated with PBS; B, larvae treated Tricyclazole; C, Pyroquilon; D, Carpropamid; E, Fenoxanil; F, Glyphosate; and G, Sulcotrione. Grains from larvae treated with carpropamid, fenoxanil and glyphosate showed less melanin intensity and encapsulation compared to PBS, while grains from the tricyclazole and pyroquilon group showed a slight increase in melanin intensity. Solid arrow indicates grains while dotted arrow indicates encapsulation surrounding the grains. Panel H shows the number and total grain size in larvae treated with melanin inhibitors. No significant difference in number and size of grains was observed between the treated and control group (Log-rank, *P* >.05). This figure appears in color in the online version of this article and in black and white in the printed version.

Previously, we have discovered that melanization of *M. mycetomatis* was inhibited by DHN inhibitors TCZ, PYR, CAR and FNX and pyomelanin inhibitor SCT, but not DOPA melanin inhibitor GLY *in vitro*.^12^ To determine if these melanin inhibitors were also able to inhibit the melanization of grains *in vivo*, they were tested in our *M. mycetomatis* infected *G. mellonella* grain model. At 72 h post-infection, a dark brown melanized grain is observed in the control group when larvae were treated with PBS (Fig. 2A). When larvae were treated with 5 mg/kg of PYR, all grains were melanized, and the melanization appeared to be slightly more intense compared to the PBS control group (Fig. 2C, Table 1). When larvae were treated with 5 mg/kg of TCZ, CAR or FNX, a mixed appearance of melanized and non-melanized grains were noted (Fig. 2B, D and E). In TCZ, 71% of the grains were melanized while 29% were not (Table 1). Similar to grains observed in the PYR treated group, melanization in grains seen in the TCZ treated group was also more intense compared to the control group. In the CAR treated group, 70% of the grains were melanized and 30% were not (Table 1). While in the FNX group, only 25% of grains were melanized, while 75% were not (Table 1). Melanization in grains found in CAR treated larvae was similar to that of the control group, while grains from FNX appeared to be less melanized. In all melanized grains, capsules surrounding the grains were observed.

**Table 1.** Melanization in grains and larvae survival when treated with melanin inhibitors either with and without itraconazole in an *in vivo M. mycetomatis* infected *G. mellonella* model.

	Grain melanization (%)		
Inhibitors	Melanized	Non-melanized	Larvae survival
Single treatment			
Infected control	100	0	
Tricyclazole	71	29	Decrease
Carpropamid	70	30	Increase
Fenoxanil	25	75	Increase
Pyroquilon	100	0	No difference at 5 mg Decrease at 50 mg
Glyphosate	50	50	No difference
Sulcotrione	100	0	No difference
Carpropamid + Glyphosate + Sulcotrione	100	0	Increase
			
Combination Treatment			
Infected control	100	0	
Itraconazole	88.5	11.5	No difference
Carpropamid + Itraconazole	100	0	Increase
Glyphosate + Itraconazole	84	16	No difference
Carpropamid + Glyphosate + Itraconazole	68	33	Increase

In contrast, where grains were not melanized, the absence of a capsule was noted. When larvae were treated with 5 mg/kg of DOPA-melanin inhibitor GLY, 50% of grains were non-melanized grains while the other 50% were melanized (Fig. 2F, Table 1). Where melanized, the intensity in the melanized grains were less compared to the control group. This indicated that melanization of the grains was inhibited by GLY to a certain extent. When treated with 5 mg/kg of pyomelanin inhibitor SCT, all grains appeared to be melanized (Fig. 2G, Table 1). The melanin intensity in SCT treated grains is similar to those from the control group. This observation did not come as a surprise due to pyomelanins nature of being a secreted pigment. Pyomelanin is most likely not bound to the surface of grains as DHN- and DOPA-melanin is. Although inhibition of the DHN- or the DOPA-melanin pathway using melanin inhibitors is sufficient to obtain non-melanized grains inside *G. mellonella*, none of the melanin inhibitors tested resulted in any significant change to the number and the total size of grains found in the larvae (Fig. 2H).

**Figure 3.**
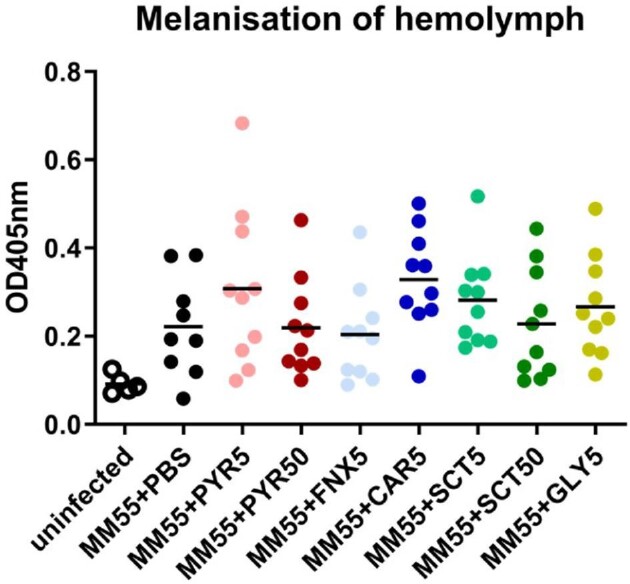
Melanization in *Galleria mellonella* larvae infected with *Madurella mycetomatis* isolate MM55 and treated with melanin inhibitors. Melanization of the hemolymph was demonstrated by measuring the OD_405nm_ of the hemolymph. All infected larvae treated with melanin inhibitors melanized and melanization rate is comparable or higher than the infected PBS control group. This indicated that the melanin inhibitors did not interfere with melanin production in larvae. PYR5, pyroquilon 5 mg/kg; PYR50, pyroquilon 50 mg/kg; FNX5, fenoxanil 5 mg/kg; CAR5, carpropamid 5 mg/kg; SCT5, sulcotrione 5 mg/kg; SCT50, sulcotrione 50 mg/kg; GLY5, glyphosate 5 mg/kg. The infected control group is referred as MM55 + PBS groups in this figure.

Since melanization is also part of *G. mellonella*’s immune response against invading pathogens, we investigated the effects of these melanin inhibitors and if these inhibitors could interfere with larvae melanization and then in turn affect grain melanization. Hemolymph was collected and measured from various groups of larvae either infected or treated with melanin inhibitors. From Fig. 3, a very low absorbance rate was noted in non-infected larvae. A higher absorbance rate was observed in the infected larvae groups, both treated or not. No significant difference in absorbance rate was observed between the infected control and the treated group (Mann--Whitney, *P*-values ranging from.08 to.9). This difference in absorbance rate between uninfected larvae and those infected and treated indicated that the inhibitors used did not affect the melanization of *G. mellonella.*

### Enhanced survival in larvae treated when DHN-melanin is inhibited with CAR

**Figure 4.**
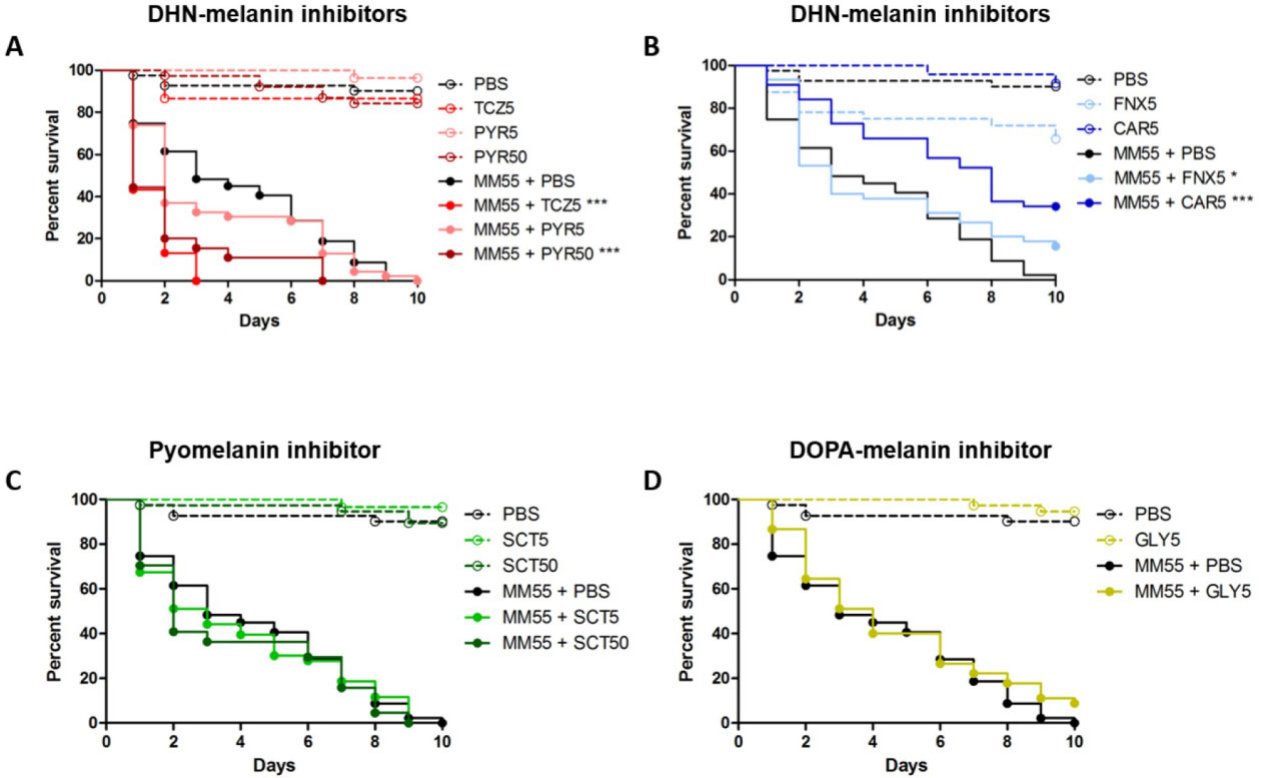
Toxicity of the inhibitors and the survival of *M. mycetomatis* isolate MM55 infected larvae treated with the inhibitors. Only fenoxanil was observed to be toxic for the larvae. Tricyclazole and pyroquilon at 5 and 50 mg/kg respectively increased larvae mortality, while carpropamid and fenoxanil both at 5 mg/kg increased larvae survival. TCZ, tricyclazole; PYR, pyroquilon; CAR, carpropamid; FNX, fenoxanil; SCT, sulcotrione; GLY, glyphosate. Significant survival was displayed as ^*^ (*P* < .05), or ^***^ (*P* < .001). The infected control group is referred as MM55 + PBS groups in this figure. Data shown here is the compilation of three individual experiments. This figure appears in color in the online version of this article and in black and white in the printed version.

To determine if inhibiting grain melanization influences the overall survival of the *G. mellonella* larvae, we monitored the survival of *M. mycetomatis* infected larvae treated with melanin inhibitors for 10 days. No significant difference in larvae survival was observed between the non-infected treatment and the non-infected control groups. At a concentration of 5 mg/kg TCZ and 50 mg/kg PYR, a significantly enhanced mortality was noted in *M. mycetomatis* infected larvae as shown in Fig. 4A (Log-Rank, *P* < .001 for both conditions). The median time to death was one day for both TCZ and PYR treated larvae, compared to five days for PBS treated larvae. This observed mortality could be explained by the toxicity of melanin-precursors or by-products produced when THR in the DHN-melanin pathway is inhibited. Indeed, juglone, one of the by-products, is highly toxic when administered to *G. mellonella* larvae at concentrations of 0.4, 4 and 40 μg/larvae (Fig. 1, Fig. S1). In contrast, when SCD in the DHN melanin pathway was inhibited with 5 mg/kg CAR or 5 mg/kg FNX, a significant increase in larvae survival was observed in both groups (Log-Rank, *P* < .001 and *P* < .05 respectively). However, FNX was found to be toxic to *G. mellonella* larvae. At 5 mg/kg FNX, larvae survival was decreased by 35% compared to the PBS treated controls (Log-Rank, *P* = .01) (Fig. 4B).

**Figure 5.**
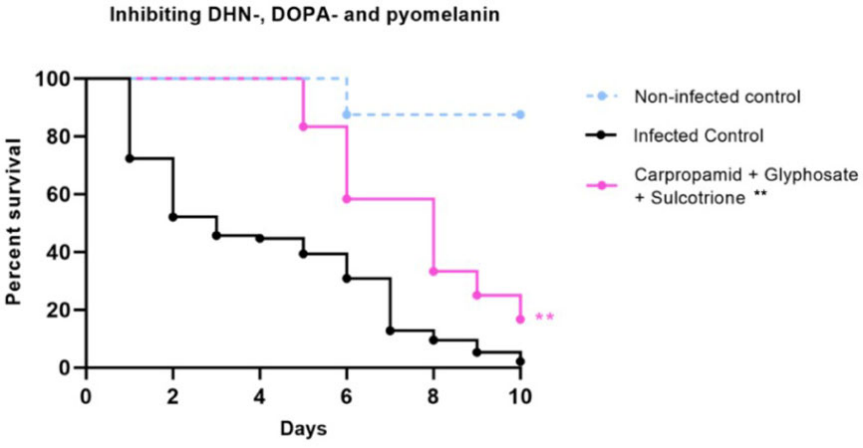
Survival of *M. mycetomatis* isolate MM55 infected larvae treated with DHN-, DOPA- and pyomelanin inhibitors carpropamid, glyphosate and sulcotrione. Larvae survival over 10 days after infection and treatment with DHN-, DOPA- and pyomelanin inhibitors carpropamid, glyphosate and sulcotrione. An increased in survival was observed compared to the infected control group. Significant survival was displayed as ^**^ (Log-Rank, *P* = .004). Data shown here is the compilation of three individual experiments. This figure appears in color in the online version of this article and in black and white in the printed version.

When inhibiting with 5 mg/kg of DOPA-melanin inhibitor GLY, no difference in larvae survival was observed (Fig. 4D). Pyomelanin inhibitor SCT also showed no difference in survival both 5 mg/kg and 50 mg/kg (Fig. 4C). To determine what happens when all three melanin biosynthesis pathways are inhibited, larvae were treated with 5 mg/kg of CAR, GLY and SCT. All grains were observed to be melanized, and a significant increase in larvae survival was observed (Log-Rank, *P* =.0038) (Fig. 5).

### Combination of Carpropamid, Glyphosate and Itraconazole enhanced larvae survival *in vivo*

**Figure 6.**
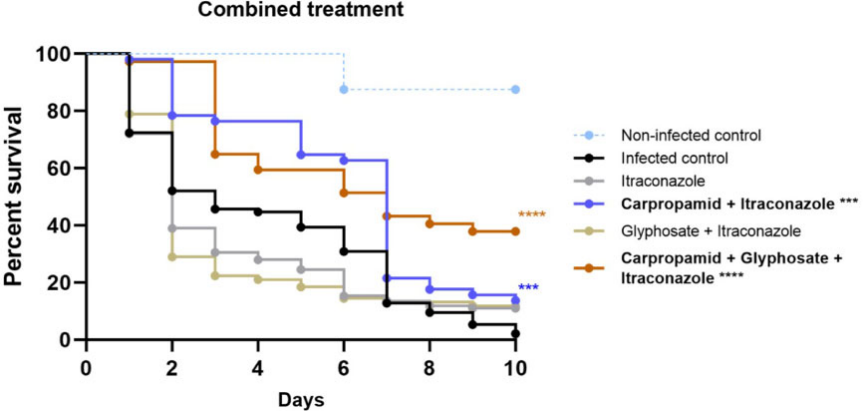
Survival of *M. mycetomatis* isolate MM55 infected larvae treated with combination of melanin inhibitors and itraconazole. Larvae survival over 10 days after infection and treatment with melanin inhibitors and itraconazole. An increased in survival was observed when larvae were treated with the combination of carpropamid and itraconazole; and the combination of carpropamid, glyphosate and itraconazole. Significant survival was displayed as ^***^ (Log-Rank, *P* < .001), or ^****^ (Log-Rank, *P* < .0001). Data shown here is the compilation of three individual experiments. This figure appears in color in the online version of this article and in black and white in the printed version.

**Figure 7.**
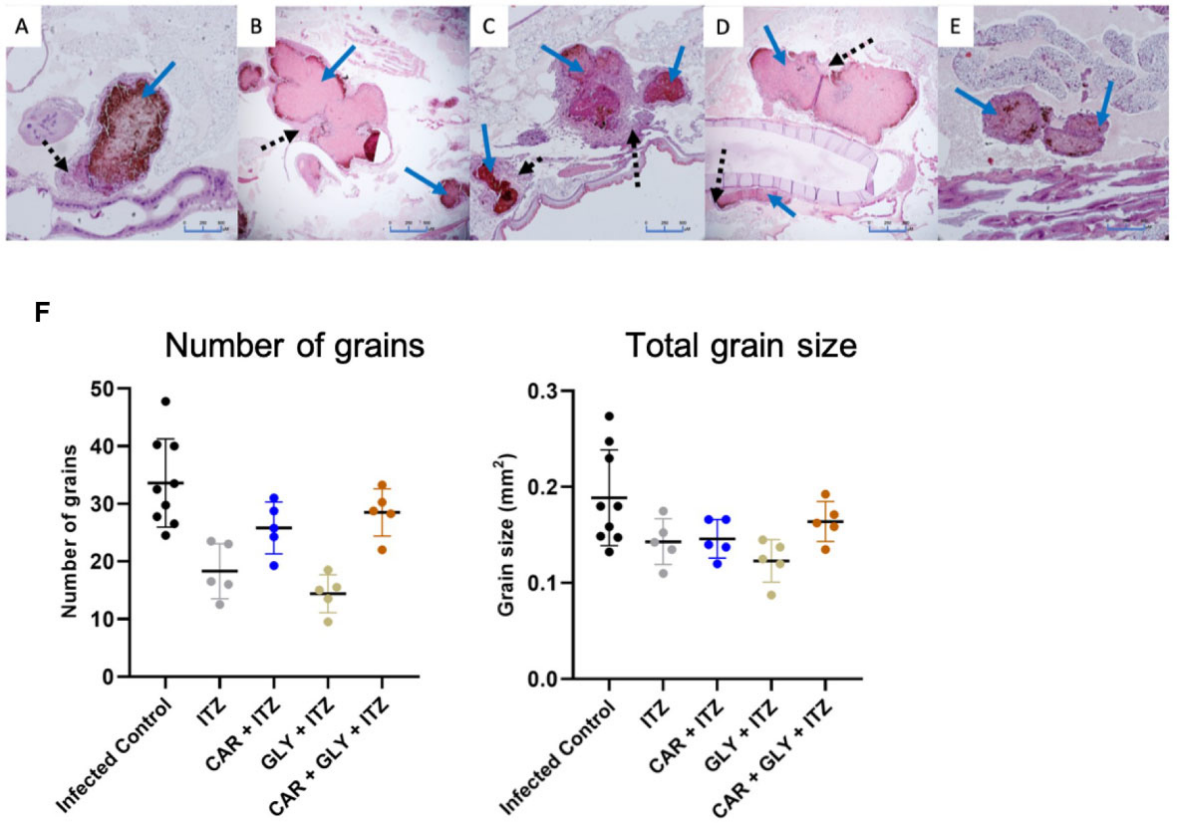
Fungal burden of larvae treated with combination of melanin inhibitors and itraconazole. Histopathological sections of larvae treated with different melanin inhibitors and sacrificed 72 h after inoculation stained with H&E to demonstrate the presence of fungal grains and encapsulation indicated by arrows. Panel A, Infected control group treated with PBS; B, Itraconazole; C, Carpropamid and Itraconazole; D, Glyphosate and Itraconazole; E, Carpropamid, glyphosate and itraconazole. All grains from the carpropamid and itraconazole treated larvae are melanized and encapsulated compared to larvae treated with the other combinations. Panel F shows the number and total grain size in combined treated larvae treated. No significant difference in number and size of grains was observed between the treated and control group.

Since treatment with DHN-melanin inhibitor CAR resulted in non-melanized grains and was able to prolong survival with no noticeable toxicity in the larvae, we wondered if this would also result in grains that are more penetrable to antifungals. To investigate this hypothesis, we treated *M. mycetomatis* infected larvae with a combination of melanin inhibitors and 5.7 mg/kg itraconazole (ITZ). Combining 5 mg/kg CAR with ITZ resulted in a significant increase in larvae survival compared to the untreated control group and itraconazole-treated group (Log-Rank, *P* = .0001 and *P* < .0001 respectively) (Fig. 6, table 1). On the tenth day post-infection, 17% of larvae treated with CAR and ITZ survived compared to only 2% in the control group (Table 1). All grains observed in the CAR and ITZ treatment group appeared to be melanized (Fig. 7C). The grains were more melanized and encapsulated compared to the PBS control group and the ITZ only group (Fig. 7A and B). Treatment with DOPA inhibitor GLY resulted in more non-melanized grains compared to CAR, but as observed in Fig. 6, the combination of 5 mg/kg GLY with ITZ did not result in any significant difference in larvae survival. Approximately 84% of the grains in this group was observed to be melanized, and the other 16% was not (Fig. 7D, Table 1). We next treated larvae with the combination of CAR, GLY and ITZ to determine if inhibiting both DHN- and DOPA-melanin biosynthesis could increase larvae survival compared to only inhibiting one melanin biosynthesis pathway at a time. This combination resulted in a significantly enhanced larvae survival (Log-Rank, *P* < .0001) (Fig. 6). Approximately 40% of larvae survived compared to the control group. A mix of melanized and non-melanized grains were observed in this treatment group; 68% were melanized while 33% were not (Fig. 7E, Table 1). Non-melanized grains were void of capsules while on the melanized grains, melanization and encapsulation were similar to that of the control group. None of the combination treatments tested resulted in any significant change to the number and the total size of grains found in the larvae (Fig. 7F).

## Discussion

In this study, we have demonstrated that inhibiting DHN- and DOPA-melanin with CAR, FNX, and GLY resulted in non-melanized grains *in vivo.* Inhibiting DHN-melanin with CAR and FNX resulted in an increase in larvae survival. Furthermore, combination treatment with CAR, GLY and ITZ also resulted in enhanced larvae survival in *M. mycetomatis* infected larvae.

Previously, we have found no noticeable DOPA-melanin inhibition with GLY in *M. mycetomatis in vitro*.^12^ However, non-melanized grains were found in larvae treated with GLY in this study. There are three plausible explanations for the appearance of non-melanized grains *in vivo*. First, *M. mycetomatis* grains are melanized by DOPA-melanin produced by *G. mellonella.*^42^ Second, treatment with GLY could have caused an indirect inhibition of fungal systems or other melanin pathways such as DHN-melanin or other eumelanin resulting in the loss of melanin production *in vivo.*^34,43–45^ Third, *M. mycetomatis* forms DOPA-melanin but only under certain growth conditions, here for example, *in vivo*. Insects in the Lepidoptera family such as *G. mellonella* produces several types of melanin, including DOPA-melanin and Dopamine-melanin.^46^ The DOPA-melanin biosynthesis pathway proposed in insects is similar to the one in fungi.^42,43,46–49^ GLY is known to inhibit auto-polymerization of L-dopa in the fungus *Cryptococcus neoformans*,^34^ this could be similar in *G. mellonella* larvae. In a recent study published in BioRXiv, the authors demonstrated that GLY could inhibit *G. mellonella* melanization *ex vivo.*^50^ However, in our *in vivo* study, we have demonstrated that GLY did not affect melanization in larvae hemolymph. Therefore, it is unlikely that the lack of melanization in *M. mycetomatis* grains in *G. mellonella* larvae was due to the interference of the insect DOPA-melanin pathway by GLY. Furthermore, GLY's well-known target - 5-enolpyruvoylshikimate 3-phosphate (EPSP) synthase is found in plants, fungi and bacteria, but not in insects.^51,52^ EPSP synthase was confirmed to be present in the genome of *M. mycetomatis* (accession number: KXX73034) and we have also identified orthologues of *GSK3* kinase and two transcription factors Bzp4, Usv101 (accession numbers: KXX73138.1, KXX77836.1 and KXX74113.1) required for the induction of regulating DOPA-melanin synthesis in *C. neoformans* in the genome of *M. mycetomatis.*^22,53^ Our findings hinted that the DOPA-melanin synthesis pathway is present in *M. mycetomatis*. We therefore hypothesize that *M. mycetomatis* produces DOPA-melanin only under certain growth conditions. This finding is not unusual as the production and utilization of different melanin types can vary between different fungi morphological phases. In *S. schenckii*, DHN-melanin is produced in its conidial form while DOPA-melanin is produced in its hyphal form under certain conditions.^54,55^ When supplemented with L-DOPA, *S. schenckii* conidia were able to produce and incorporate DOPA-melanin in its cell wall when DHN melanin was inhibited.^54^ In *A. fumigatus*, DHN-melanin is produced in conidia while pyomelanin in hyphae,^14,56^ and both are differentially expressed under certain growth conditions *in vitro.*^57^

DHN-melanin inhibition with TCZ and PYR resulted in strongly melanized grains *in vivo* and an increase in larvae mortality. On the contrary, DHN-melanin inhibitors CAR and FNX, which inhibit another enzyme in the DHN-melanin pathway, inhibited melanization in grains, but increased larvae survival. In the DHN-melanin pathway, TCZ and PYR inhibit tetrahydroxynaphthalene reductase (THR). While THR is found twice in the DHN melanin synthesis pathway, the majority of TCZ and PYR's activity is against the second THR in this pathway.^58–61^ The second THR was found to be present in *M. mycetomatis* genome (accession number: KXX75671.1). Inhibiting THR results in the accumulation of melanin precursors and by-products such as flaviolin and juglone.^60,62^ These by-products are brown pigmented^59,61,63^ and could be trapped within the grain cement material when produced, thus explaining the increased melanization in grains found in TCZ and PYR treated larvae. This increase in larvae mortality was not due to the toxicity of either TCZ and PYR since we have shown that these inhibitors are not toxic to larvae. Instead, we hypothesize that this high mortality was due to the toxicity of juglone. Juglone is able to induce oxidative and genotoxic stress in *G. mellonella*^64^ and is also toxic to *G. mellonella* as discovered here. Tricyclazole has been previously tested on *M. mycetomatis* and other black-grain causing mycetoma causing agents *in vitro*; our findings show a decrease in expansion growth.^12,36^ Juglone's toxicity outweighs its antifungal properties, as demonstrated here and in *A. fumigatus, A. flavus, C. albicans* and *Fusarium spp*.^65,66^ In this case, it was clear that inhibiting DHN-melanin biosynthesis with CAR was a better approach since a significantly enhanced survival was observed over all CAR treated larvae.

To determine if the therapeutic value of itraconazole (ITZ) would increase by inhibiting DHN- and/or DOPA-melanin biosynthesis, combination treatments were performed in *G. mellonella*. When ITZ was combined with either DHN-inhibitor CAR or DOPA-melanin-inhibitor GLY, an increase in melanized grains was found compared to a single treatment with either CAR or GLY. Combining ITZ and CAR led to the increase in larvae survival while combining ITZ with GLY however, did not. Our data suggested that larvae survival was independent of grain melanization. There are two plausible explanations for the increase in grain melanization. First, inducing stress to the fungus by inhibiting ergosterol biosynthesis with melanin inhibitors and ITZ resulted in the upregulation of alternative melanin biosynthesis pathways. For example, when DHN-melanin biosynthesis is inhibited, DOPA- or other melanin biosynthesis will be induced by this stress and vice versa, resulting in more melanin production. Second, capsules form by ITZ prevents CAR or GLY from reaching fungal grains to inhibit melanin production. In mycetoma, treatment with ITZ has been associated with thick encapsulation surrounding fungal grains in humans.^7^ Similar encapsulation was found surrounding *M. mycetomatis* grains in larvae treated with ITZ and CAR here in this study. This also suggested that ITZ associated encapsulation was formed very quickly in larvae just before the second treatment of CAR or GLY can reach and penetrate the grains. Our findings here hinted towards a complex regulation of the melanin biosynthesis pathways and the different roles between DHN- and DOPA-melanin which we have yet to understand. While the different roles of DHN- and DOPA-melanin in *M. mycetomatis* cannot yet be established, the positive impact of the combination of melanin inhibitors and ITZ in treatment is clear and should be further investigated to evaluate its potential. Because the thick encapsulation caused by ITZ hampers grain accessibility, other antifungals without this trait should also be evaluated to determine their efficacy in this combination treatment.

With the controversy surrounding GLY's safety,^67,68^ it is never in our intention to consider or even use it for treatment. The role of melanin in mycetoma is still not very well understood, however, it is certain that it protects *M. mycetomatis* and increases resistance to antifungals. As melanin inhibition is a new avenue for drug target and development, our findings here have proved that melanin inhibition should be further explored together with other, better melanin inhibitors. Further studies in mammalian models is also crucial to investigate the potential use of melanin inhibitors in therapy, and since most common black-grain eumycetoma causing agents produce similar types of melanin, this approach can be explored for all black-grain mycetoma cases. Melanin inhibition strategy can also be explored in actinomycetoma since pigmented grains are also present. In this study, we suggest that different types of melanin are utilized in *M. mycetomatis in vitro* versus *in vivo.* Although the role of the different melanin types are not fully understood, it is highly probable that each melanin types play a different role in *M. mycetomatis* virulence. Melanin inhibition as an alternative treatment concept should be further evaluated in mycetoma treatment.

## Supplementary Material

myac003_Supplemental_Figure
