# Supplementary figures and images for: Inhibiting DHN- and DOPA-melanin biosynthesis pathway increased the therapeutic value of itraconazole in Madurella mycetomatis infected Galleria mellonella
Source: Med Mycol. 2022 Jan 22;60(2):myac003. doi: 10.1093/mmy/myac003 (PMC9295015; doi:10.1093/mmy/myac003)

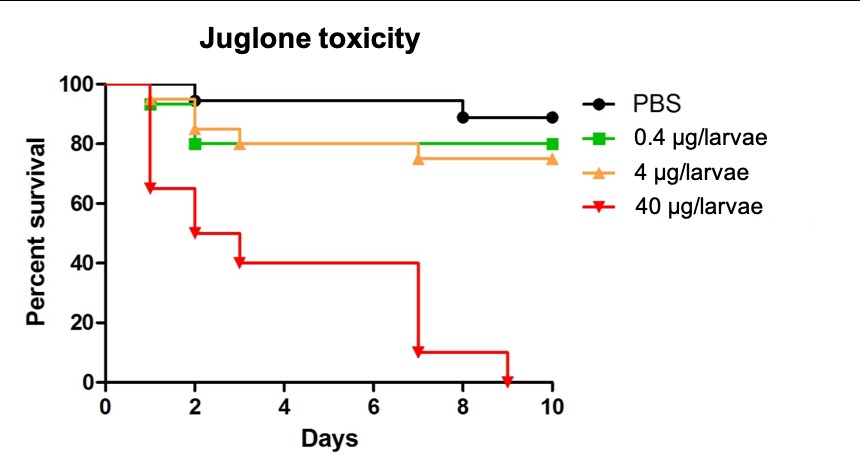

Supplement: myac003_Supplemental_Figure [file myac003_supplemental_figure.jpeg]
